# Prediction of Lower Flammability Limits for Binary Hydrocarbon Gases by Quantitative Structure—Property Relationship Approach

**DOI:** 10.3390/molecules24040748

**Published:** 2019-02-19

**Authors:** Yong Pan, Xianke Ji, Li Ding, Juncheng Jiang

**Affiliations:** Jiangsu Key Laboratory of Hazardous Chemicals Safety and Control, College of Safety Science and Engineering, Nanjing Tech University, Nanjing 210009, China; 1090997173@njtech.edu.cn (X.J.); dilydily@163.com (L.D.); ypnjut@126.com (J.J.)

**Keywords:** lower flammability limit, binary hydrocarbon gases, mixture descriptors, quantitative structure–property relationship (QSPR)

## Abstract

The lower flammability limit (LFL) is one of the most important parameters for evaluating the fire and explosion hazards of flammable gases or vapors. This study proposed quantitative structure−property relationship (QSPR) models to predict the LFL of binary hydrocarbon gases from their molecular structures. Twelve different mixing rules were employed to derive mixture descriptors for describing the structures characteristics of a series of 181 binary hydrocarbon mixtures. Genetic algorithm (GA)-based multiple linear regression (MLR) was used to select the most statistically effective mixture descriptors on the LFL of binary hydrocarbon gases. A total of 12 multilinear models were obtained based on the different mathematical formulas. The best model, issued from the norm of the molar contribution formula, was achieved as a six-parameter model. The best model was then rigorously validated using multiple strategies and further extensively compared to the previously published model. The results demonstrated the robustness, validity, and satisfactory predictivity of the proposed model. The applicability domain (AD) of the model was defined as well. The proposed best model would be expected to present an alternative to predict the LFL values of existing or new binary hydrocarbon gases, and provide some guidance for prioritizing the design of safer blended gases with desired properties.

## 1. Introduction

The lower flammability limit (LFL), is defined as the minimum volume percentage concentration in air in which a flammable substance can create a fire or explosion when an ignition source appears [1]. LFL is an important parameter widely used to reflect the flammability hazard of gases and vapors in the practical industry process. The LFL values of chemicals can be obtained through experimental tests, literature inquiry and other methods [2,3,4,5]. However, although the experimental tests are the most reliable, accurate and main source of the LFL data used in practice, the lower flammability limits of various mixtures are always infrequently reported, since the properties of the mixtures are closely related to their compositions and ratios, which are rather difficult to test one by one. Therefore, it is of great significance to develop theoretical models for predicting the LFL for mixtures.

There are two main theoretical methods for predicting the lower flammability limit of flammable mixtures in the literature. One is the theoretical derivation model based on the Le Chatelier equation. Zhao et al. [6] measured the flammability limits of binary saturated/unsaturated hydrocarbon mixtures. The validity of Le Chatelier’s law was also evaluated for those fuel mixtures, and a modification of this law was developed from the experimental data. The other is the empirical model based on physicochemical parameters. Ma [7] proposed a methodology based on thermal balance for estimating the flammability limits of a mixture. This method is equivalent to Le Chatelier’s rule but has increased flexibility in dealing with various fuel/oxygen/diluents combinations. Although these methods could provide acceptable prediction results for LFL of mixtures, they also have some obvious defects, especially the limited applicability domain of the models which are usually only applicable to mixtures with specific compositions.

A current trend in predicting the physicochemical properties is the use of the quantitative structure–properties relationship (QSPR) method. QSPR is a mathematical method that relates the properties of interest to the molecular structures of chemicals which are represented by a variety of molecular descriptors. In many cases, QSPR techniques have been successfully used for the prediction of different properties for pure chemicals, which have been extensively reviewed elsewhere [8,9,10,11,12,13,14]. However, only a few studies have been completed on QSPR models for mixtures [15,16,17,18,19,20,21,22,23], due to the complexity of the structure description of the mixtures. In most studies, the molecular descriptors for each pure chemical were combined by mole-weighted averaging to derive mixture descriptors. These mixture descriptors were then correlated to the property of the studied mixtures. As for the QSPR studies for predicting the lower flammability limit of the mixtures, the involved studies are scarce. Recently, Wang et al. [18] developed three QSPR models based on the same dataset but with three different external validation strategies to predict the lower flammability limits of blended gases. The same mixtures descriptors calculated by mole weighted average using the individual descriptor values and mole fraction of each component were employed.

The present study aimed to develop new QSPR models for the lower flammability limit of binary hydrocarbon gases and to evaluate the potential of various mathematical formulas of mixture descriptors for the development of such models for predicting this property. Also, the most reliable QSPR model for predicting the lower flammability limit of binary hydrocarbon gases, which are of industrial importance, would be selected and proposed from only their molecular structures.

## 2. Materials and Methods

### 2.1. Data Set

In this study, an extended database obtained by gathering additional data from the literature to enlarge the data set employed by Wang et al. [24] was used in order to extend the diversity of the chemicals involved in the mixtures. The new dataset consisting of 181 binary hydrocarbon gases which was taken from the literature [6,25,26,27,28]. A complete list of the detailed compositions of mixtures as well as the LFL values was presented in Table S1 in the Supporting Information . It comprises 10 different pure chemicals, which are methane, ethane, propane, butane, isobutane, ethylene, propylene, butylene, butadiene and acetylene. The lower flammability limit values range from 1.65 to 4.71 vol.% while mole fractions vary from 0.1 to 0.9. As is well known, with a larger dataset a better predictive model could be developed, but it is rather difficult to find a larger set of LFL data for hydrocarbon gases mixtures in the publicly available literature in terms of chemical diversity.

### 2.2. Molecular Descriptors for Pure Chemicals

Molecular descriptors are defined as numerical characteristics related to the molecular structures of a chemical component [29,30]. In the present work, the molecular descriptors calculated by the Dragon 6.0 software (Talete S.r.l., Milano, Italy) were used to characterize the structural features of pure hydrocarbon [31]. Dragon 6.0 is a sophisticated program for the calculation of molecular descriptors which can calculate 29 types and 4885 kinds of molecular descriptors which were presented in Table 1. 

Firstly, the structures of all pure chemicals were drawn in 2D ChemDraw (PerkinElmer, Waltham, MA, USA). Then, the drawn structures were optimized using MM+ molecular mechanics force field by the 3D ChemDraw based on the minimum energy molecular geometries optimized. Subsequently, geometry optimization was carried out by Materials Studio 2017 software (Dassault Systèmes, San Diego, CA, USA) based on the AM1 semi-empirical method to obtain the stable conformation with the minimum energy. Afterwards, all the energy minimized structures were ported to Dragon 6.0 software to calculate the corresponding molecular descriptors. In all, a total of 1037 descriptors were calculated for each chemical in the dataset. 

After the calculation of molecular descriptors, the descriptors that stayed constant and near constant for all studied chemicals were removed from the descriptor pool, because those descriptors were not encoding the structural differences between chemicals that account for their different lower flammability limit values. Further reduction of the descriptor pool was attained by examining pairwise correlations between descriptors, so that only one descriptor was retained from a pair that contributed similar information (correlation coefficient > 0.90 in this study). Finally, a total set of 93 remaining descriptors that have significant contributions to the lower flammability limit was obtained.

### 2.3. Determination of Mixture Descriptors

The definition of mixture descriptors was identified as a critical parameter in existing QSPR models for mixtures, in particular for properties that can follow a nonlinear trend with the concentration of each chemical. In the previous work of Gaudin et al. [20], 12 different mixing rules (mathematical formulas) were employed to calculate the mixture descriptors for the development for QSPR models to predict the flash points of binary mixtures. Mixture descriptors D were developed by combining the molecular descriptors (*d_i_*) of each chemical, taking into account their respective mole fractions (*x_i_*). The same 12 mixing rules were tested in this study for the development of QSPR models to predict the lower flammability limits of binary hydrocarbon gases. The 12 formulas can be divided into three classes: direct combinations, deviation combinations and other combinations. The formulas were provided in Table 2.

### 2.4. Descriptor Selection and Model Development

One of the most important steps involved in QSPR studies is to select the optimal subset of descriptors that have significant contribution to the desired property. The well-known genetic algorithm (GA) is a powerful optimization method for solving this problem [32]. This algorithm is developed to search for the global optima of solutions, and it has been successfully applied to feature selection in QSPR studies. In this work, the GA- multiple linear regression (MLR) method, which is a sophisticated hybrid approach that combines GA with MLR [33], was used to find the optimal subset of descriptors that can efficiently represent the relationship between molecular structures and the LFL for binary hydrocarbon gases. The program required to perform GA-MLR in this study was written in MATLAB (MathWorks, Natick, MA, USA) M-file in our laboratory.

### 2.5. Model Validation

Model validation is of crucial importance for the developed QSPR models. In this study, various validation strategies were employed to validate the performance of the developed models for their fitness, robustness, and predictivity. First, the widely used statistical parameters, the squared correlation coefficient (*R*^2^), the root-mean-square error (RMSE), and the average absolute error (AAE) were employed for the validation of the fitness of the model. AAE and RMSE are calculated as follows:

(13) $$AAE=\frac{\sum_{i=1}^{n}\;\left|y_{i}-y_{o}\right|}{n},$$

(14) $$RMSE=\sqrt{\frac{\sum_{i=1}^{n}{\left(y_{i}-y_{o}\right)}^{2}}{n}},$$

where *y_i_* is the experimental value, *y_o_* is the predicted value, and *n* is the number of chemicals in the data set.

Second, the internal robustness of the models was evaluated by the leave-one-out (LOO) cross-validation test on the training set [34]. The outcome from such a test is a cross-validated correlation coefficient, $Q_{LOO}^{2}$, which is calculated according to the following formula:

(15) $$Q_{LOO}^{2}=1-\frac{\sum_{1}^{training}{\left(y_{i}-y_{o}\right)}^{2}}{\sum_{1}^{training}{\left(y_{i}-\bar{y}\right)}^{2}},$$

where *y_i_*, *y_o_*, and $\bar{y}$ were respectively the experimental, predicted, and mean experimental LFL values of the chemicals in the training set. 

The external validation is a significant and necessary validation method used to determine both the generalization performance and the true predictive capability of the QSPR models for new mixtures. There are three different validation strategies widely used in the literature, including “points out’’, ‘‘mixtures out’’ and ‘‘compounds out’’. ‘‘Points out’’ is the prediction of the observed property for any composition from the dataset, ‘‘Mixtures out’’ is the prediction of the observed property for novel mixtures which are absent in the training set, and ‘‘Compounds out’’ is the prediction of the observed property for mixtures formed by novel pure compounds, and these pure compounds are absent in the training set. Among these three strategies, the “Points out” strategy is applicable if and only if several mixtures with the same components with different ratios are present in the training set. However, this method is the easiest and the simplest one, and it will fully reflect the ability of models to predict existing mixtures with new composition. Thus, in this study, the external validation was carried out by randomly splitting the available data set into a training set (80% of the dataset) and an external test set (20% of the dataset) based on the “Points out” partition strategy. The training set is used for descriptor selection and model development, while the test set is used for model validation. Moreover, in order to avoid obtaining the QSPR results by chance or obtaining non-general conclusions, all the developed models were tested on a sufficiently large number of chemicals (20% of the dataset) in the test sub-sets [35]. The predictive capability of a QSPR model can be judged by an external $Q_{ext}^{2}$ which is defined as follows:

(16) $$Q_{ext}^{2}=1-\frac{\sum_{i=1}^{test}{\left(y_{i}-y_{o}\right)}^{2}}{\sum_{i=1}^{test}{\left(y_{i}-{\bar{y}}_{tr}\right)}^{2}},$$

where *y_i_* and *y_o_* were the experimental and predicted LFL values of the chemicals in the test set, and ${\bar{y}}_{tr}$ was the mean experimental LFL values of the chemicals in the training set. Moreover, the *r*_m_^2^ metrics introduced by Roy et al. [36] cold also be used for validation of regression-based QSPR models.

Finally, the Y-randomization test was also employed to further ensure the robustness of the involved model. The Y-randomization test is also a widely used technique to ensure the model robustness [37], whereby the performances of the original model in data description are compared to that of models built for permuted (randomly shuffled) response, based on the original descriptor pool and the original model building procedure. The process is repeated 50–100 times. If all the randomized generated models have much lower *R*^2^ values than that of the original model as expected, and the difference between *R*^2^ of the original model and the mean highest random (mhr) *R*^2^ is higher than 2.3 standard deviation (SD) for significance on the 1% level, higher than 3 SD for the 0.1% level, etc, one may reasonably conclude that there is no chance correlation in the model development.

### 2.6. Applicability Domain

According to the Organisation for Economic Co-operation and Development (OECD) principle 3 [38], an applicability domain (AD) should be defined when an acceptable QSPR model is proposed. It is a valid range to determine whether the new mixtures can be reliably predicted by the developed model. There are many existing methods for the definition of AD, such as range based, distance based, geometrical based and probability based methods [39]. In this study, the definition of AD is based on the leverage values and the cross-validated standardized residuals, which can be depicted in the Williams plot. It is a scatter plot presenting the investigated chemicals in two dimensions. If the leverage value (*h_i_*) of a mixture is higher than the standard leverage value (h*), it could be regarded as being out of the application range of the model. If the cross-validated standardized residual is greater than 3 standard deviation units of the model, the mixture is classified as an outlier. In other words, all the data should be in the rectangular area of h* and 3 standard deviation units and the points on the boundary could be considered acceptable, *h_i_* and h* are calculated as follows:

(17) $$h_{i}=x_{i}^{T}{\left(X^{T}X\right)}^{-1}x_{i}\;\left(i=1,2,3,\ldots ,\;n\right),$$

where *x_i_* is the descriptor vector of the considered mixture and *X* is the model matrix derived from the training set descriptor values.

(18) $$h^{*}=\frac{3\left(q+1\right)}{n},$$

where *q* is the number of mixture descriptors used in the model, and *n* is the data size of the training set.

## 3. Results and Discussion

### 3.1. Comparisons of the Twelve Developed Models

By performing the GA-MLR procedure on the training set based on different mathematical formulas, the different optimum subsets of mixture descriptors were achieved. The types and definitions of these descriptors were presented in Tables S2–S13. A total of 12 different new QSPR models for the lower flammability limit of binary hydrocarbon gases were developed, which were presented as Equations (S1)–(S12) in the Supporting Information. The performances of all the 12 developed models were presented in Table S14.

To evaluate the potential of the 12 involved formulas for the model development for predicting LFL, it should be noted that the “deviation formulas” aimed to describe the deviation of the property from the linear contributions of the two components with their respective concentrations. Thus, the three corresponding formulas (mol_dev, sqr_mol_dev, and mol_dev_sqr) were not tested directly on the LFL but on the difference between the actual experimental LFL values and the linear contribution of the experimental LFL values of the pure hydrocarbons weighted by their respective mole fraction in the mixtures. However, all the three deviation formulas (Table S14) failed in accessing reliable models, with *R*^2^ = 0.484 for mol_dev, *R*^2^ = 0.525 for sqr_mol_dev and *R*^2^ = 0.360 for mol_dev_sqr, respectively.

**Figure 1:**
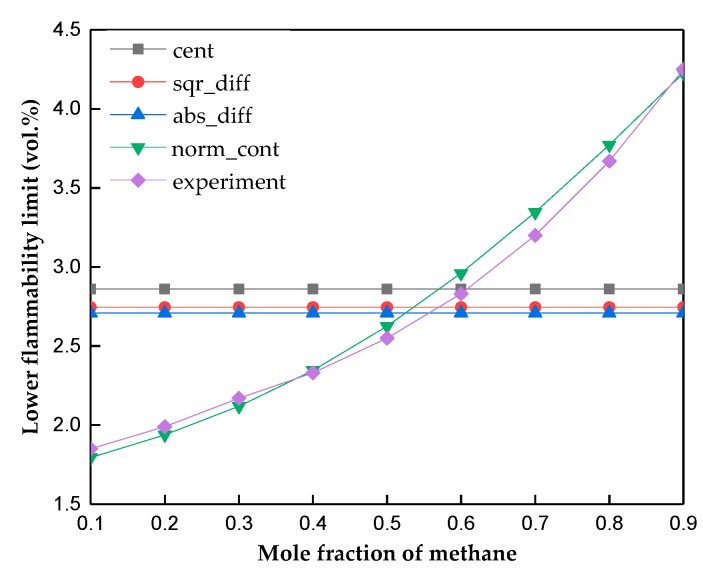
Experimental and predicted lower flammability limit (LFL) values for methane/butylene mixtures.

Afterwards, as for the “other combinations” (Equations (10)–(12) in Table 2), the capacity of the model issued from each combination to reproduce qualitatively the shapes of the mixture profiles of the data set was examined. The results showed that the cent, sqr_diff and abs_diff may be less pertinent. The reasons could be contributed to the fact that all the above-mentioned three formulas do not consider the mole fractions, while the properties including lower flammability limit varies with the composition of the mixtures. For instance, the same LFL values for the whole range of concentration for the methane/butylene mixture were provided for the cent, sqr_diff and abs_diff formulas, as shown in Figure 1. 

**Figure 2:**
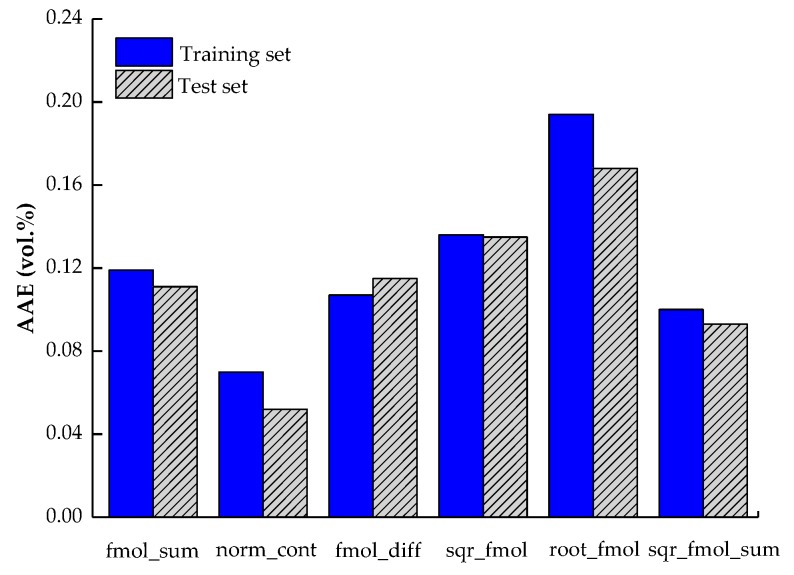
Performance comparisons of the QSPR models for the LFLs of mixtures depending upon the types of mixture descriptors.

As for the remaining six “direct combinations”, which were directly used to develop models for the prediction of LFL, better results were obtained. Meanwhile, among the six “direct combinations”, some resulting models demonstrated a much better predictive capability than others in terms of AAE values for both training and test sets, as shown in Figure 2. For example, the models based on root_fmol, sqr_fmol, and fmol_sum presented the highest errors in prediction with AAE being of 0.194 vol%, 0.136 vol%, and 0.119 vol %, respectively. The best model was obtained based on the norm of the molar contribution (norm_cont), for which the AAE value is being of 0.052 vol% for the test set. This model has a better generalization performance and predictive capability, with the $Q_{ext}^{2}$ value being of 0.988. In conclusion, one out of the 12 developed models was selected as the best model from a statistical point of view: norm_cont, which presented the best and satisfactory performances.

### 3.2. Details of the Best Model

On the basis of the previous analysis, the model developed based on the norm of the molar contribution formula (norm_cont) was selected as the best model for the prediction of lower flammability limits for binary hydrocarbon gases. The types and definitions of these descriptors are presented in Table 3. The corresponding best model is presented as the following:

(19) $$\begin{array}{c} LFL=2.720-0.567\times \sqrt{{\left(x_{1}RBN_{1}\right)}^{2}+{\left(x_{2}RBN_{2}\right)}^{2}}-4.661\times \\ \sqrt{{\left(x_{1}MAXDP_{1}\right)}^{2}+{\left(x_{2}MAXDP_{2}\right)}^{2}}+0.642\times \sqrt{{\left(x_{1}Psi\_i\_0_{1}\right)}^{2}+{\left(x_{2}Psi\_i\_0_{2}\right)}^{2}}+0.544\times \\ \sqrt{{\left(x_{1}SpMax4\_Bh{\left(e\right)}_{1}\right)}^{2}+{\left(x_{2}SpMax4\_Bh{\left(e\right)}_{2}\right)}^{2}}-2.056\times \sqrt{{\left(x_{1}Mor24u_{1}\right)}^{2}+{\left(x_{2}Mor24u_{2}\right)}^{2}}-\\ 23.689\times \sqrt{{\left(x_{1}Mor16m_{1}\right)}^{2}+{\left(x_{2}Mor16m_{2}\right)}^{2}},\\ R^{2}=0.964,\;s=0.138,\;F=606.440,\;n=145 \end{array}$$

where *R*^2^ is the squared correlation coefficient, *s* is the standard error of the model, *F* is the value of the F-test, and *n* is the number of data in the training set.

The mean effect (*ME*) percentage is used to describe the significant effects of the selected descriptors on the LFL, which is defined as follows:

(20) $$ME_{j}^{J}=\frac{\sum_{i=1}^{n}D_{ij}\times a_{j}}{\sum_{j=1}^{J}\left|\sum_{i=1}^{n}D_{ij}\times a_{j}\right|}\times 100,$$

The *ME* values of these descriptors were presented in Table 3. The symbol (positive or negative) of ME represents the trend of the impact of each descriptor on the LFL. Also, if the absolute value of ME is larger, it indicates that the descriptor is more important. As can be seen from Table 3, Psi_i_0 descriptor has the most significant influence on LFL. In addition, the relative importance and contribution of each descriptor in the model was determined and ranked based on the ME values: Psi_i_0 > Mor16m > MAXDP > SpMax4_Bh(e) > Mor24u > RBN.

Among the six descriptors, RBN is a constitutional index, which is the number of bonds that allow free rotation around themselves. These bonds are any single bond, not in a ring, bound to a nonterminal heavy atom. RBN explicitly takes into account the role of rings in a molecule. It can be determined by the *N_nt_* (the number of nonterminal freely rotatable bonds) and *n_r_* (the number of single bonds in any nonaromatic ring). The larger *N_nt_* and *n_r_* are, the lower LFL will be [30]. MAXDP is the maximal electrotopological positive variation, which is related to the total number and the type of the atoms. It plays a negative role on the LFL [29,30]. Psi_i_0 is the intrinsic state pseudoconnectivity index. The second letter *i* in the Psi_i_0 shows that this index is derived from the intrinsic state I, and the 0 is the type of the index. The intrinsic state depending on the type of the considered atom reflects the possible influence partitioning of non-σ electron. The smaller the partitioning of the electron influence, the more available are the valence electrons for intermolecular interactions [30]. SpMax4_Bh(e) is a burden eigenvalue descriptor, which is defined as eigenvalues of a modified connectivity matrix (burden matrix B) [30]. The diagonal elements are roughly proportional to the electronegativity of the atoms. The larger the electronegativity of the atoms are, the larger the SpMax4_Bh(e) value will be, which would lead to an increase in the LFL and a lower hazard. Both Mor24u and Mor16m are the 3D-MoRSE descriptors, which can be calculated by various weighting schemes or different physicochemical properties. The more values are chosen, the finer the resolution in the representation of the molecule. They have a negative impact on the LFL [30].

In conclusion, all the six employed descriptors are correlated with the simple structural features (the number of bonds, number and type of the atoms, et al.), the 3D structural characteristics as well as molecule’s electrostatic property of the mixtures. Therefore, the overall LFL property of binary hydrocarbon gases can be mainly explained by their electrostatic and steric effects.

**Figure 3:**
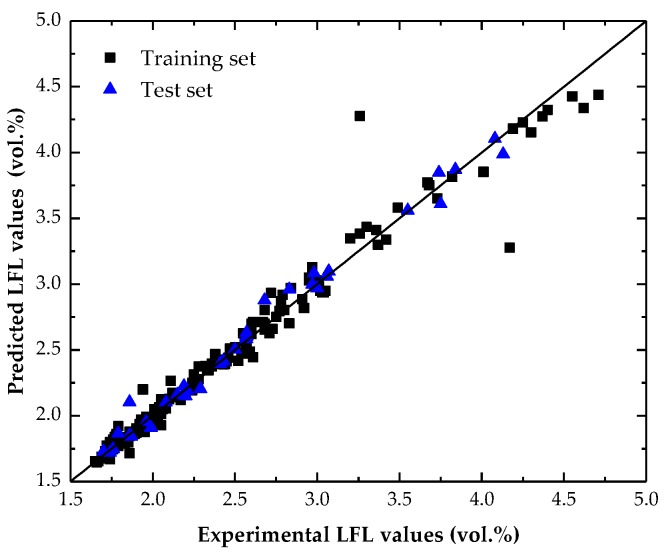
Correlation between the predicted and experimental LFL values for both the training and test sets.

The predicted LFL values of the model are presented in Table S1 in the Supporting Information . A plot of the predicted LFL values versus the experimental ones for both the training and test sets is shown in Figure 3. The obtained results presented in Figure 2 showed that the resulting AAE values for both training and test sets are within the experimental error of LFL determination, which is around ±0.1 vol% [10]. Also, both the AAE and RMSE values were not only low but also as similar as possible for the training and external test sets, which suggests both predictive capability (low values) and generalization performance (similar values) of the proposed model.

### 3.3. Model Stability Validation

**Figure 4:**
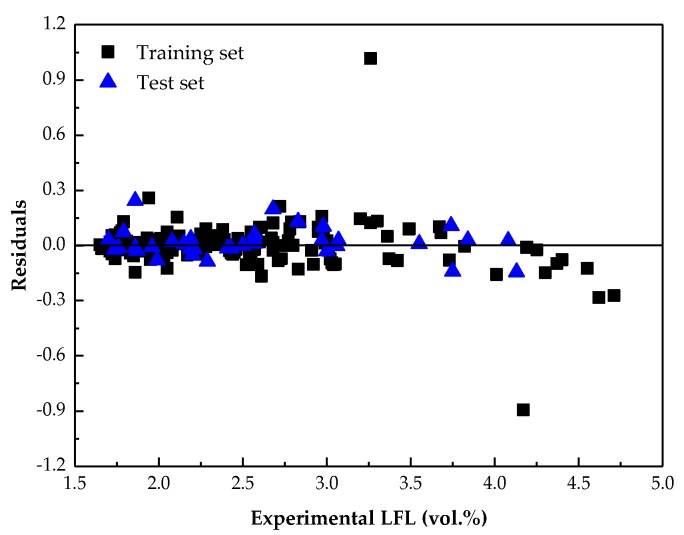
Plot of the residuals vs. the experimental LFL values for the best model.

The proposed model was then tested for chance correlation to further analyze the model stability. The Y-randomization test method was employed to ensure the robustness of the model. It was performed 100 times on the training set. The resulted maximum, minimum, and average values of the obtained random *R*^2^ were 0.133, 0.011, and 0.040, respectively, while the value of SD is 0.021. The resulted maximum, minimum, and average values of the obtained highest random *R*^2^ were 0.133, 0.011, and 0.040, respectively, while the value of SD is 0.021. The difference between *R*^2^ of the original model and the mean highest random *R*^2^ resulted in a distance of 44 SD, which is much higher than 3 SD and showed obvious statistical significance of the proposed model. As expected, all the models generated had produced low *R*^2^ values, which were much lower than the one calculated when the dependent variables were not scrambled. That is to say, only the correct dependent variables can be used to generate reasonable models. Thus, it could be reasonably concluded that chance correlation had little or even no effect in the presented model. Also, as can be seen from the predicted residual distributions of the model as shown in Figure 4, the prediction residuals are randomly distributed on both sides of the baseline, which indicated that there are no systematic errors in the model development. It can be reasonably concluded that the best model is valid and can be effectively used to predict the lower flammability limits of binary hydrocarbon gases. 

### 3.4. Applicability Domain of the Best Model

**Figure 5:**
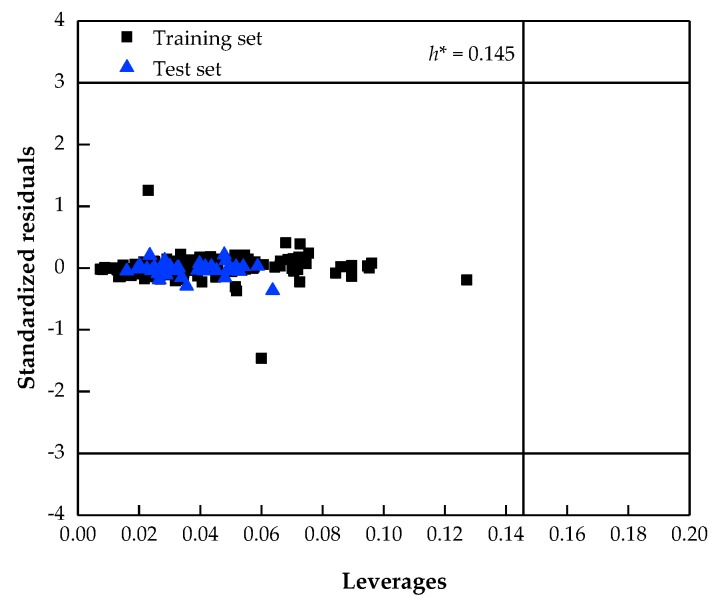
The Williams plot describing the applicability domain of the best model (h*=0.145).

The Williams plot for the best model is showed in Figure 5. From this plot, the applicability domain is established inside a squared area within ±3 standard deviations and a leverage threshold h* of 0.145. Only when a mixture lies within the domain, the predicted LFL value could be considered reliable. As can be seen from Figure 5, all the values in the dataset are within the AD area. However, it should be stated that there is also a limitation of the AD of the model in terms of chemical diversity, since only 10 different pure chemicals are composed in the studied dataset, although it is rather difficult to find a further larger set of LFL data for hydrocarbon gases mixtures in the open literatures containing more different pure hydrocarbons.

### 3.5. Comparison with Previous Works

General comparisons should be considered between the presented model and previous ones, since the various models had been developed based on different dataset and different methods. 

Compared to the Le Chatelier’s rule as well as Ma’s [7] methodology, which is equivalent to Le Chatelier's rule, the presented model can be conveniently used to predict the LFL of unknown hydrocarbon mixtures solely from the molecular structures without requiring any extra information on physicochemical properties. The use of the models of Le Chatelier’s rule requires extra data on needed physicochemical properties such as the LFL of pure chemicals, and if only one of the needed properties is missing, the calculation cannot be performed. In contrast, because only theoretical descriptors derived solely from the molecular structure is involved, the presented model would theoretically be used to reliably predict the LFL for any hydrocarbon mixtures belonging to its applicability domain. Moreover, these employed theoretical descriptors in the presented model have definite physical meanings, which are useful for probing the physicochemical information that has a significant contribution to the LFL property of binary hydrocarbon gases. In regards to Le Chatelier’s rule as well as Ma’s [7] methodology, because the estimated results are highly dependent on the accuracy of the input physicochemical properties, any errors introduced by initial measurements will be passed on to the estimated results. In addition, all flammability data are still dependent on the flame temperature or flame structure. Both will be difficult to predict using this theory.

The detailed comparisons between the best model and works of Wang et al. [24] are presented as follows. Wang et al. [24] developed three linear QSPR models between the LFL and the quantum chemical descriptors of a diverse set of blended gases. The comparisons of statistical parameters between the presented model and the models by Wang et al. [24] are showed in Table 4. As can be seen from Table 4, the presented model is obviously better in terms of both *R*^2^ and $Q_{ext}^{2}$ than the models of work [24], which showed better goodness of fit and robustness of the presented model. Moreover, the presented model is developed based on a larger number of mixtures in the dataset (181 vs. 86), and also more mixtures are employed in the test set for model external validation (36 vs. 18/19). However, two more different external validation strategies were employed to evaluate the true predictive ability of the generated models the work of Wang et al. [24] than that in this work. Regarding the input parameters used in the models, both models employed the theoretical descriptors which can be directly calculated from the molecular structures. Moreover, these theoretical descriptors have definite physical meanings, which are useful to probe the physicochemical information that has significant contribution to the LFL property of mixtures. However, the discussion of the descriptor interpretation is missing in Wang et al.’s work. Regarding the applicability efficiency of the models, all the descriptors selected in the presented model are Dragon descriptors, which could be freely accessible from the online MOLE db—Molecular Descriptors Data Base [40] comprised of 1124 molecular descriptors for 234,773 chemicals, while the quantum chemical descriptors employed in work [24] are more complicated and professional knowledge and software are needed for calculation.

## 4. Conclusions

In this study, a series of mixture descriptors have been tested in order to achieve reliable QSPR models for predicting the lower flammability limits of binary hydrocarbon gases. To the best of our knowledge, the largest existing database of lower flammability limits for mixtures was employed for modeling. A total of 12 different mixing rules was used to calculate mixture descriptors for representing the structure characteristics of hydrocarbon mixtures. The best resulting model was obtained with the norm of the molar contribution formula, as nonlinear effects were observed on the LFL with concentration. The results of rigorous model validation showed the satisfactory goodness of fit, internal robustness and predictive capability of the presented model. When comparing the results of the model to those of the previously published one, it showed that the presented model possesses some obvious superiority despite of the different composition of the studied datasets. Thus, it can be reasonably concluded that the proposed model would be expected to be valid and reliably used to predict the LFL of hydrocarbon mixtures with an accuracy that can approach the accuracy of experimental LFL determination. Additionally, this model would be expected to provide an alternative for predicting the LFL values of existing or new binary hydrocarbon gases belonging to its applicability domain.

## Figures and Tables

**Table 1:** The types and numbers of descriptors calculated by Dragon 6.0 software.

Type	Number	Type	Number
Constitutional descriptors	43	Ring descriptors	32
Topological indices	75	Walk and path counts	46
Connectivity indices	37	Information indices	48
2D matrix-based descriptors	550	2D autocorrelations	213
Burden eigenvalues	96	P_VSA-like descriptors	45
ETA indices	23	Edge adjacency indices	324
Geometrical descriptors	38	3D matrix-based descriptors	90
3D autocorrelations	80	RDF descriptors	210
3D-MoRSE descriptors	224	WHIM descriptors	114
GETAWAY descriptors	273	Randic molecular profiles	41
Functional group counts	154	Atom-centered fragments	115
Atom-type E-state indices	170	CATS 2D	150
2D Atom Pairs	1596	3D Atom Pairs	36
Charge descriptors	15	Molecular properties	20
Drug-like indices	27		

P_VSA: van der Waals surface area having a property P; ETA: extended topochemical atom; RDF: radial distribution function; MoRSE: molecule representation of structures based on electron diffraction; WHIM: weighted holistic invariant molecular; CATS: chemically advanced template search.

**Table 2:** Twelve formulas used for calculation of mixture descriptors.

Mixing Rule	Binary Mixtures	Equation
**Direct combinations**		
Molar contribution (fmol_sum)	$D=x_{1}d_{1}+x_{2}d_{2}$	(1)
Norm of the molar contribution (norm_cont)	$D=\sqrt{{\left(x_{1}d_{1}\right)}^{2}+{\left(x_{2}d_{2}\right)}^{2}}$	(2)
Weighted difference (fmol_diff)	$D=\left|x_{1}d_{1}-x_{2}d_{2}\right|$	(3)
Square mole fraction (sqr_fmol)	$D=x_{1}^{2}d_{1}+x_{2}^{2}d_{2}$	(4)
Square-root mole fraction (root_fmol)	$D=\sqrt{x_{1}}d_{1}+\sqrt{x_{2}}d_{2}$	(5)
Square molar contribution (sqr_fmol_sum)	$D={\left(x_{1}d_{1}+x_{2}d_{2}\right)}^{2}$	(6)
**Deviation combinations**		
mol_dev	D=(1−Δx)Δd	(7)
sqr_mol_dev	$D=\left(1-\Delta x^{2}\right)\Delta d$	(8)
mol_dev_sqr	$D={\left(1-\Delta x\right)}^{2}\Delta d$	(9)
**Other combinations**		
Centroid approach (cent)	$D=\frac{d_{1}+d_{2}}{2}$	(10)
Square of the difference (sqr_diff)	$D={\left(d_{1}-d_{2}\right)}^{2}$	(11)
Absolute difference (abs_diff)	$D=\left|d_{1}-d_{2}\right|$	(12)

**Table 3:** Descriptors selected for the best model for prediction of LFL.

Descriptor	Type	Definition	*ME* Value
RBN	Constitutional indices	Number of rotatable bonds	−5.0%
MAXDP	Topological indices	Maximal electrotopological positive variation	−19.4%
Psi_i_0	Topological indices	Intrinsic state pseudoconnectivity index—type 0	32.5%
SpMax4_Bh(e)	Burden eigenvalues	Largest eigenvalue n. 4 of Burden matrix weighted by Sanderson electronegativity	14.5%
Mor24u	3D-MoRSE descriptors	Signal 24/unweighted	−5.7%
Mor16m	3D-MoRSE descriptors	Signal 16/weighted by mass	−22.9%

*ME*: Mean effect.

**Table 4:** Comparison of statistical parameters between the presented model and previous ones.

	Model	Training set					Test set				
		*R*^2^	$Q_{LOO}^{2}$	AAE	RMSE	*n*	*R*^2^	$Q_{ext}^{2}$	AAE	RMSE	*n*
Wang et al. work [24] ^a^	Model 1	0.916	0.901	0.041	0.053	68	0.935	0.932	0.040	0.051	18
	Model 2	0.916	0.896	0.045	0.058	68	0.932	0.964	0.022	0.029	18
	Model 3	0.923	0.905	0.041	0.054	67	0.944	0.899	0.042	0.048	19
This work	The best model	0.964	0.964	0.070	0.137	145	0.988	0.988	0.052	0.077	36

^a^ Both the AAE and RMSE values of the models of Wang et al. work are for log LFL while which of this work are for LFL.

## Supplementary Materials

The following are available online: Table S1: A complete list of the detailed compositions of mixtures as well as the experimental and predicted LFL values (the best model), Table S2: Descriptors selected for the prediction model based on fmol_sum mixing rule, Table S3: Descriptors selected for the prediction model based on norm_cont mixing rule, Table S4: Descriptors selected for the prediction model based on fmol_diff mixing rule, Table S5: Descriptors selected for the prediction model based on sqr_fmol mixing rule, Table S6: Descriptors selected for the prediction model based on root_fmol mixing rule, Table S7: Descriptors selected for the prediction model based on sqr_fmol_sum mixing rule, Table S8: Descriptors selected for the prediction model based on mol_dev mixing rule, Table S9: Descriptors selected for the prediction model based on sqr_mol_dev mixing rule, Table S10: Descriptors selected for the prediction model based on mol_dev_sqr mixing rule, Table S11: Descriptors selected for the prediction model based on cent mixing rule, Table S12: Descriptors selected for the prediction model based on sqr_diff mixing rule, Table S13: Descriptors selected for the prediction model based on abs_diff mixing rule, Table S14: Performances of all the 12 developed models based on mixture descriptors issued from the 12 mixing rules. Equations (S1)–(S12): The 12 different QSPR models for the lower flammability limit of binary hydrocarbon gases based on different mixing rules.
