# Supplementary material for: Prediction of Lower Flammability Limits for Binary Hydrocarbon Gases by Quantitative Structure—Property Relationship Approach
Source: Molecules. 2019 Feb 19;24(4):748. doi: 10.3390/molecules24040748 (PMC6413142; doi:10.3390/molecules24040748)
Supplement: Supplementary file 1 [file molecules-24-00748-s001.pdf]

Prediction of Lower Flammability Limits for Binary Hydrocarbon Gases by Quantitative Structure–Property Relationship Approach

Yong Pan *, Xianke Ji, Juncheng Jiang and Li Ding

SUPPLEMENTARY MATERIALS

Table S1. A complete list of the detailed compositions of mixtures as well as the experimental and predicted LFL values (the best model)

No.	Component A		Component B		Exp. LFL (vol%)	Pre. LFL (vol%)	Status	Ref.
	Chemical	Molar fraction	Chemical	Molar fraction				
1	propane	0.2	isobutane	0.8	1.73	1.70	Training	26
2	propane	0.4	isobutane	0.6	1.79	1.92	Training	26
3	propane	0.8	isobutane	0.2	1.94	2.20	Training	26
4	methane	0.125	butane	0.875	1.86	1.72	Training	6
5	methane	0.25	butane	0.75	2.05	1.93	Training	6
6	methane	0.5	butane	0.5	2.56	2.57	Training	6
7	methane	0.625	butane	0.375	2.95	3.03	Training	6
8	methane	0.75	butane	0.25	3.49	3.58	Training	6
9	methane	0.875	butane	0.125	4.19	4.18	Training	6
10	methane	0.125	propane	0.875	2.24	2.25	Training	6
11	methane	0.25	propane	0.75	2.45	2.41	Training	6
12	methane	0.375	propane	0.625	2.73	2.66	Training	6
13	methane	0.625	propane	0.375	3.36	3.41	Training	6
14	methane	0.875	propane	0.125	4.40	4.32	Training	6
15	methane	0.125	acetylene	0.875	2.61	2.44	Training	6
16	methane	0.25	acetylene	0.75	2.83	2.70	Training	6
17	methane	0.375	acetylene	0.625	3.00	3.03	Training	6
18	methane	0.5	acetylene	0.5	3.26	3.39	Training	6
19	methane	0.625	acetylene	0.375	3.68	3.75	Training	6
20	methane	0.875	acetylene	0.125	4.55	4.43	Training	6
21	ethylene	0.125	propylene	0.875	2.32	2.38	Training	6
22	ethylene	0.25	propylene	0.75	2.43	2.39	Training	6
23	ethylene	0.5	propylene	0.5	2.52	2.42	Training	6
24	ethylene	0.625	propylene	0.375	2.55	2.47	Training	6
25	ethylene	0.875	propylene	0.125	2.75	2.75	Training	6
26	ethylene	0.125	acetylene	0.875	2.44	2.39	Training	6
27	ethylene	0.375	acetylene	0.625	2.58	2.61	Training	6
28	ethylene	0.5	acetylene	0.5	2.61	2.71	Training	6
29	ethylene	0.625	acetylene	0.375	2.68	2.80	Training	6
30	ethylene	0.875	acetylene	0.125	2.72	2.93	Training	6
31	methane	0.3	ethane	0.7	4.17	3.28	Training	27
32	methane	0.8	ethane	0.2	3.26	4.28	Training	27
33	methane	0.125	ethylene	0.875	3.01	2.99	Training	6
34	methane	0.375	ethylene	0.625	3.37	3.30	Training	6
35	methane	0.625	ethylene	0.375	4.01	3.85	Training	6
36	methane	0.75	ethylene	0.25	4.30	4.15	Training	6
37	methane	0.875	ethylene	0.125	4.71	4.44	Training	6
38	methane	0.1	propylene	0.9	2.42	2.39	Training	28

No.	Component A		Component B		Exp. LFL (vol%)	Pre. LFL (vol%)	Status	Ref.
	Chemical	Molar fraction	Chemical	Molar fraction				
39	methane	0.2	propylene	0.8	2.59	2.49	Training	28
40	methane	0.3	propylene	0.7	2.71	2.63	Training	28
41	methane	0.4	propylene	0.6	2.92	2.82	Training	28
42	methane	0.6	propylene	0.4	3.42	3.34	Training	28
43	methane	0.7	propylene	0.3	3.73	3.65	Training	28
44	methane	0.75	propylene	0.25	3.82	3.82	Training	25
45	methane	0.9	propylene	0.1	4.62	4.34	Training	28
46	methane	0.1	butylene	0.9	1.85	1.80	Training	28
47	methane	0.2	butylene	0.8	1.99	1.94	Training	28
48	methane	0.3	butylene	0.7	2.17	2.12	Training	28
49	methane	0.4	butylene	0.6	2.33	2.35	Training	28
50	methane	0.5	butylene	0.5	2.55	2.63	Training	28
51	methane	0.7	butylene	0.3	3.20	3.35	Training	28
52	methane	0.8	butylene	0.2	3.67	3.77	Training	28
53	methane	0.9	butylene	0.1	4.25	4.23	Training	28
54	methane	0.1	butadiene	0.9	1.90	1.87	Training	28
55	methane	0.2	butadiene	0.8	2.05	2.01	Training	28
56	methane	0.3	butadiene	0.7	2.24	2.19	Training	28
57	methane	0.4	butadiene	0.6	2.43	2.42	Training	28
58	methane	0.5	butadiene	0.5	2.67	2.71	Training	28
59	methane	0.6	butadiene	0.4	2.95	3.05	Training	28
60	methane	0.7	butadiene	0.3	3.30	3.43	Training	28
61	methane	0.9	butadiene	0.1	4.37	4.27	Training	28
62	ethane	0.1	ethylene	0.9	3.05	2.95	Training	28
63	ethane	0.2	ethylene	0.8	3.04	2.94	Training	28
64	ethane	0.3	ethylene	0.7	3.03	2.94	Training	28
65	ethane	0.4	ethylene	0.6	3.02	2.95	Training	28
66	ethane	0.6	ethylene	0.4	2.99	3.00	Training	28
67	ethane	0.7	ethylene	0.3	2.98	3.04	Training	28
68	ethane	0.9	ethylene	0.1	2.97	3.13	Training	28
69	ethane	0.1	propylene	0.9	2.28	2.37	Training	28
70	ethane	0.2	propylene	0.8	2.36	2.40	Training	28
71	ethane	0.4	propylene	0.6	2.46	2.44	Training	28
72	ethane	0.5	propylene	0.5	2.54	2.48	Training	28
73	ethane	0.6	propylene	0.4	2.57	2.55	Training	28
74	ethane	0.7	propylene	0.3	2.68	2.65	Training	28
75	ethane	0.8	propylene	0.2	2.77	2.80	Training	28
76	ethane	0.9	propylene	0.1	2.84	2.97	Training	28
77	ethane	0.1	butylene	0.9	1.78	1.78	Training	28
78	ethane	0.2	butylene	0.8	1.86	1.88	Training	28
79	ethane	0.3	butylene	0.7	1.93	1.97	Training	28

No.	Component A		Component B		Exp. LFL (vol%)	Pre. LFL (vol%)	Status	Ref.
	Chemical	Molar fraction	Chemical	Molar fraction				
80	ethane	0.4	butylene	0.6	2.04	2.06	Training	28
81	ethane	0.6	butylene	0.4	2.28	2.28	Training	28
82	ethane	0.7	butylene	0.3	2.43	2.43	Training	28
83	ethane	0.8	butylene	0.2	2.60	2.62	Training	28
84	ethane	0.9	butylene	0.1	2.78	2.87	Training	28
85	ethane	0.1	butadiene	0.9	1.87	1.86	Training	28
86	ethane	0.3	butadiene	0.7	2.01	2.05	Training	28
87	ethane	0.4	butadiene	0.6	2.14	2.15	Training	28
88	ethane	0.5	butadiene	0.5	2.25	2.26	Training	28
89	ethane	0.6	butadiene	0.4	2.36	2.37	Training	28
90	ethane	0.7	butadiene	0.3	2.50	2.52	Training	28
91	ethane	0.8	butadiene	0.2	2.60	2.70	Training	28
92	ethane	0.9	butadiene	0.1	2.79	2.92	Training	28
93	propane	0.1	ethylene	0.9	2.91	2.89	Training	28
94	propane	0.2	ethylene	0.8	2.77	2.79	Training	28
95	propane	0.3	ethylene	0.7	2.68	2.70	Training	28
96	propane	0.5	ethylene	0.5	2.47	2.51	Training	28
97	propane	0.6	ethylene	0.4	2.39	2.42	Training	28
98	propane	0.7	ethylene	0.3	2.34	2.35	Training	28
99	propane	0.8	ethylene	0.2	2.28	2.28	Training	28
100	propane	0.9	ethylene	0.1	2.22	2.21	Training	28
101	propane	0.1	butylene	0.9	1.79	1.77	Training	28
102	propane	0.2	butylene	0.8	1.82	1.82	Training	28
103	propane	0.4	butylene	0.6	1.89	1.86	Training	28
104	propane	0.5	butylene	0.5	1.95	1.88	Training	28
105	propane	0.7	butylene	0.3	2.03	1.97	Training	28
106	propane	0.8	butylene	0.2	2.08	2.06	Training	28
107	propane	0.9	butylene	0.1	2.10	2.13	Training	28
108	propane	0.1	butadiene	0.9	1.86	1.85	Training	28
109	propane	0.2	butadiene	0.8	1.90	1.90	Training	28
110	propane	0.3	butadiene	0.7	1.92	1.94	Training	28
111	propane	0.4	butadiene	0.6	1.96	1.96	Training	28
112	propane	0.5	butadiene	0.5	2.01	1.97	Training	28
113	propane	0.6	butadiene	0.4	2.03	2.01	Training	28
114	propane	0.7	butadiene	0.3	2.06	2.05	Training	28
115	propane	0.9	butadiene	0.1	2.12	2.14	Training	28
116	butane	0.1	ethylene	0.9	2.80	2.80	Training	28
117	butane	0.3	ethylene	0.7	2.38	2.47	Training	28
118	butane	0.4	ethylene	0.6	2.25	2.31	Training	28
119	butane	0.5	ethylene	0.5	2.12	2.17	Training	28
120	butane	0.6	ethylene	0.4	2.03	2.04	Training	28

No.	Component A		Component B		Exp. LFL (vol%)	Pre. LFL (vol%)	Status	Ref.
	Chemical	Molar fraction	Chemical	Molar fraction				
121	butane	0.7	ethylene	0.3	1.92	1.91	Training	28
122	butane	0.8	ethylene	0.2	1.83	1.79	Training	28
123	butane	0.9	ethylene	0.1	1.74	1.67	Training	28
124	butane	0.1	propylene	0.9	2.11	2.26	Training	28
125	butane	0.2	propylene	0.8	2.05	2.12	Training	28
126	butane	0.3	propylene	0.7	1.96	1.99	Training	28
127	butane	0.4	propylene	0.6	1.91	1.90	Training	28
128	butane	0.6	propylene	0.4	1.80	1.81	Training	28
129	butane	0.7	propylene	0.3	1.76	1.78	Training	28
130	butane	0.8	propylene	0.2	1.71	1.73	Training	28
131	butane	0.9	propylene	0.1	1.65	1.66	Training	28
132	butane	0.1	butylene	0.9	1.76	1.74	Training	28
133	butane	0.4	butylene	0.6	1.73	1.69	Training	28
134	butane	0.5	butylene	0.5	1.72	1.68	Training	28
135	butane	0.6	butylene	0.4	1.71	1.68	Training	28
136	butane	0.7	butylene	0.3	1.70	1.69	Training	28
137	butane	0.8	butylene	0.2	1.69	1.69	Training	28
138	butane	0.9	butylene	0.1	1.66	1.64	Training	28
139	butane	0.1	butadiene	0.9	1.81	1.84	Training	28
140	butane	0.3	butadiene	0.7	1.78	1.86	Training	28
141	butane	0.4	butadiene	0.6	1.77	1.84	Training	28
142	butane	0.5	butadiene	0.5	1.76	1.82	Training	28
143	butane	0.6	butadiene	0.4	1.74	1.80	Training	28
144	butane	0.7	butadiene	0.3	1.72	1.77	Training	28
145	butane	0.9	butadiene	0.1	1.67	1.66	Training	28
146	propane	0.6	isobutane	0.4	1.86	2.10	Test	26
147	methane	0.375	butane	0.625	2.29	2.20	Test	6
148	methane	0.5	propane	0.5	2.97	3.00	Test	6
149	methane	0.75	propane	0.25	3.84	3.87	Test	6
150	methane	0.75	acetylene	0.25	4.08	4.11	Test	6
151	ethylene	0.375	propylene	0.625	2.41	2.40	Test	6
152	ethylene	0.75	propylene	0.25	2.57	2.58	Test	25
153	ethylene	0.25	acetylene	0.75	2.50	2.50	Test	6
154	ethylene	0.75	acetylene	0.25	2.68	2.88	Test	6
155	methane	0.5	ethane	0.5	3.75	3.61	Test	27
156	methane	0.25	ethylene	0.75	3.07	3.10	Test	25
157	methane	0.5	ethylene	0.5	3.55	3.56	Test	25
158	methane	0.5	propylene	0.5	3.06	3.06	Test	25
159	methane	0.8	propylene	0.2	4.13	3.99	Test	28
160	methane	0.6	butylene	0.4	2.83	2.96	Test	28
161	methane	0.8	butadiene	0.2	3.74	3.85	Test	28

No.	Component A		Component B		Exp. LFL (vol%)	Pre. LFL (vol%)	Status	Ref.
	Chemical	Molar fraction	Chemical	Molar fraction				
162	ethane	0.5	ethylene	0.5	3.00	2.97	Test	28
163	ethane	0.8	ethylene	0.2	2.98	3.08	Test	28
164	ethane	0.3	propylene	0.7	2.43	2.42	Test	28
165	ethane	0.5	butylene	0.5	2.15	2.16	Test	28
166	ethane	0.2	butadiene	0.8	1.96	1.95	Test	28
167	propane	0.4	ethylene	0.6	2.57	2.60	Test	28
168	propane	0.3	propylene	0.7	2.19	2.23	Test	28
169	propane	0.4	propylene	0.6	2.19	2.18	Test	28
170	propane	0.5	propylene	0.5	2.20	2.15	Test	28
171	propane	0.8	propylene	0.2	2.20	2.19	Test	28
172	propane	0.9	propylene	0.1	2.21	2.19	Test	28
173	propane	0.3	butylene	0.7	1.86	1.85	Test	28
174	propane	0.6	butylene	0.4	1.99	1.91	Test	28
175	propane	0.8	butadiene	0.2	2.08	2.10	Test	28
176	butane	0.2	ethylene	0.8	2.57	2.63	Test	28
177	butane	0.5	propylene	0.5	1.87	1.84	Test	28
178	butane	0.2	butylene	0.8	1.76	1.75	Test	28
179	butane	0.3	butylene	0.7	1.74	1.72	Test	28
180	butane	0.2	butadiene	0.8	1.79	1.87	Test	28
181	butane	0.8	butadiene	0.2	1.70	1.73	Test	28

1. The molar contribution (fmol_sum) mixing rule

$$LFL = -15.465X_1 - 0.702X_2 - 0.827X_3 + 0.409X_4 - 34.957X_5 + 5.442 \quad (S1)$$

Table S2. Descriptors selected for the prediction model based on fmol_sum mixing rule.

Variable	Descriptor	Type	Definition
X_1	TDB02v	3D autocorrelations	3D Topological distance based descriptors - lag 2 weighted by van der Waals volume
X_2	Mor04u	3D-MoRSE descriptors	signal 04 / unweighted
X_3	Mor27u	3D-MoRSE descriptors	signal 27 / unweighted
X_4	E1p	WHIM descriptors	1st component accessibility directional WHIM index / weighted by polarizability
X_5	HATS2m	GETAWAY descriptors	leverage-weighted autocorrelation of lag 2 / weighted by mass

2. The norm of the molar contribution (norm_cont) mixing rule

$$LFL=-0.567X_1-4.661X_2+0.642X_3+0.544X_4-2.056X_5-23.689X_6+2.720 \quad (S2)$$

Table S3. Descriptors selected for the prediction model based on norm_cont mixing rule.

Variable	Descriptor	Type	Definition
X_1	RBN	Constitutional indices	number of rotatable bonds
X_2	MAXDP	Topological indices	maximal electrotopological positive variation
X_3	Psi_i_0	Topological indices	intrinsic state pseudoconnectivity index - type 0
X_4	SpMax4_Bh(e)	Burden eigenvalues	largest eigenvalue n. 4 of Burden matrix weighted by Sanderson electronegativity
X_5	Mor24u	3D-MoRSE descriptors	signal 24 / unweighted
X_6	Mor16m	3D-MoRSE descriptors	signal 16 / weighted by mass

3. The weighted difference (fmol_diff) mixing rule

$$LFL=0.240X_1-0.142X_2-0.765X_3-0.729X_4-10.234X_5+10.524X_6-29.423X_7-0.276X_8+3.780 \quad (S3)$$

Table S4. Descriptors selected for the prediction model based on fmol_diff mixing rule.

Variable	Descriptor	Type	Definition
X_1	Psi_i_0	Topological indices	intrinsic state pseudoconnectivity index - type 0
X_2	P_VSA_LogP_3	P_VSA-like descriptors	P_VSA-like on LogP, bin 3
X_3	RDF020u	RDF descriptors	Radial Distribution Function - 020 / unweighted

X_4	RDF040u	RDF descriptors	Radial Distribution Function - 040 / unweighted
X_5	Mor16u	3D-MoRSE descriptors	signal 16 / unweighted
X_6	Mor09m	3D-MoRSE descriptors	signal 09 / weighted by mass
X_7	Mor26m	3D-MoRSE descriptors	signal 26 / weighted by mass
X_8	SssCH2	Atom-type E-stat indices	Sum of ssCH2 E-states

4. The square mole fraction (sqr_fmol) mixing rule

$$LFL=-2.587X_1-1.084X_2+6.545X_3+15.349X_4+2.269 \quad (S4)$$

Table S5. Descriptors selected for the prediction model based on sqr_fmol mixing rule.

Variable	Descriptor	Type	Definition
X_1	GGI1	2D autocorrelations	topological charge index of order 1
X_2	E2u	WHIM descriptors	2nd component accessibility directional WHIM index / unweighted
X_3	P2m	WHIM descriptors	2nd component shape directional WHIM index / weighted by mass
X_4	R2p+	GETAWAY descriptors	R maximal autocorrelation of lag 2 / weighted by polarizability

5. The square-root mole fraction (root_fmol) mixing rule

$$LFL=2.318X_1-1.100X_2-0.001X_3-0.139X_4+3.946 \quad (S5)$$

Table S6. Descriptors selected for the prediction model based on root_fmol mixing rule.

Variable	Descriptor	Type	Definition
X_1	Psi_i_1s	Topological indices	intrinsic state pseudoconnectivity index - type 1s
X_2	BID	Walk and path counts	Balaban ID number
X_3	L/Bw	Geometrical descriptors	length-to-breadth ratio by WHIM
X_4	HATS3u	GETAWAY descriptors	leverage-weighted autocorrelation of lag 3 / unweighted

6. The square molar contribution (sqr_fmol_sum) mixing rule

$$LFL=-0.124X_1+0.517X_2+18.468X_3+0.371X_4-0.435X_5+3.814 \quad (S6)$$

Table S7. Descriptors selected for the prediction model based on sqr_fmol_sum mixing rule.

Variable	Descriptor	Type	Definition
X_1	AMW	Constitutional indices	average molecular weight
X_2	GATS2m	2D autocorrelations	Geary autocorrelation of lag 2 weighted by mass
X_3	VE2_G/D	3D matrix-based	average coefficient of the last eigenvector from

		descriptors	distance/distance matrix
X_4	Mor04u	3D-MoRSE descriptors	signal 04 / unweighted
X_5	ALOGP	Molecular properties	Ghose-Crippen octanol-water partition coeff. (logP)

7. Deviation combination (mol_dev) mixing rule

$$LFL=-2.390X_1+1.548X_2-0.505X_3-3.041X_4+4.225X_5-1.952X_6+2.578 \tag{S7}$$

Table S8. Descriptors selected for the prediction model based on mol_dev mixing rule.

Variable	Descriptor	Type	Definition
X_1	MAXDN	Topological indices	maximal electrotopological negative variation
X_2	MATS2m	2D autocorrelations	Moran autocorrelation of lag 2 weighted by mass
X_3	SpMax4_Bh(e)	Burden eigenvalues	largest eigenvalue n. 4 of Burden matrix weighted by Sanderson electronegativity
X_4	Mor27u	3D-MoRSE descriptors	signal 27 / unweighted
X_5	E1p	WHIM descriptors	1st component accessibility directional WHIM index / weighted by polarizability
X_6	Di	WHIM descriptors	D total accessibility index / weighted by ionization potential

8. Deviation combination (sqr_mol_dev) mixing rule

$$LFL=-1.426X_1-0.109X_2+0.971X_3-12.458X_4+2.913X_5+2.646 \tag{S8}$$

Table S9. Descriptors selected for the prediction model based on sqr_mol_dev mixing rule.

Variable	Descriptor	Type	Definition
X_1	MAXDP	Topological indices	maximal electrotopological positive variation
X_2	Psi_i_s	Topological indices	intrinsic state pseudoconnectivity index - type S
X_3	MATS2m	2D autocorrelations	Moran autocorrelation of lag 2 weighted by mass
X_4	Mor27p	3D-MoRSE descriptors	signal 27 / weighted by polarizability
X_5	E1p	WHIM descriptors	1st component accessibility directional WHIM index / weighted by polarizability

9. Deviation combination (mol_dev_sqr) mixing rule

$$LFL=-4.605X_1+0.770X_2-1.078X_3+0.427X_4+3.251X_5+2.552 \tag{S9}$$

Table S10. Descriptors selected for the prediction model based on mol_dev_sqr mixing rule.

Variable	Descriptor	Type	Definition
X_1	MAXDN	Topological indices	maximal electrotopological negative variation
X_2	BID	Walk and path counts	Balaban ID number
X_3	SpMax4_Bh(e)	Burden eigenvalues	largest eigenvalue n. 4 of Burden matrix weighted by Sanderson electronegativity
X_4	RDF020u	RDF descriptors	Radial Distribution Function - 020 / unweighted
X_5	E1e	WHIM descriptors	1st component accessibility directional WHIM index / weighted by Sanderson electronegativity

10. Centroid approach (cent) mixing rule

$$LFL=-0.043X_1-11.379X_2+1.137X_3-2.091X_4+5.471 \quad (S10)$$

Table S11. Descriptors selected for the prediction model based on cent mixing rule.

Variable	Descriptor	Type	Definition
X_1	MW	Constitutional indices	molecular weight
X_2	TDB02v	3D autocorrelations	3D Topological distance based descriptors - lag 2 weighted by van der Waals volume
X_3	Mor32u	3D-MoRSE descriptors	signal 32 / unweighted
X_4	Mor04v	3D-MoRSE descriptors	signal 04 / weighted by van der Waals volume

11. The square of the difference (sqr_diff) mixing rule

$$LFL=0.314X_1-0.142X_2-4.076X_3-5.585X_4-0.506X_5+4.502X_6+2.807 \quad (S11)$$

Table S12. Descriptors selected for the prediction model based on sqr_diff mixing rule.

Variable	Descriptor	Type	Definition
X_1	BID	Walk and path counts	Balaban ID number
X_2	RDF040u	RDF descriptors	Radial Distribution Function - 040 / unweighted
X_3	Mor15u	3D-MoRSE descriptors	signal 15 / unweighted
X_4	Mor25u	3D-MoRSE descriptors	signal 25 / unweighted
X_5	G1u	WHIM descriptors	1st component symmetry directional WHIM index / unweighted
X_6	E1p	WHIM descriptors	1st component accessibility directional WHIM index /

weighted by polarizability

12. The absolute difference (abs_diff) mixing rule

$$LFL=-0.955X_1-0.234X_2+7.846X_3-9.335X_4-0.424X_5+0.765X_6+2.827 \quad (S12)$$

Table S13. Descriptors selected for the prediction model based on abs_diff mixing rule.

Variable	Descriptor	Type	Definition
X_1	MAXDN	Topological indices	maximal electrotopological negative variation
X_2	SpMax4_Bh(e)	Burden eigenvalues	largest eigenvalue n. 4 of Burden matrix weighted by Sanderson electronegativity
X_3	TDB02v	3D autocorrelations	3D Topological distance based descriptors - lag 2 weighted by van der Waals volume
X_4	Mor20m	3D-MoRSE descriptors	signal 20 / weighted by mass
X_5	G1u	WHIM descriptors	1st component symmetry directional WHIM index / unweighted
X_6	E1e	WHIM descriptors	1st component accessibility directional WHIM index / weighted by Sanderson electronegativity

Table S14. Performances of all the 12 developed models based on mixture descriptors issued from the 12 mixing rules.

Mixing rules	Number of Descriptors	Training Set				Test Set		
		R^2	Q^2_{LOO}	AAE	RMSE	Q^2_{ext}	AAE	RMSE
**Direct combinations**								
fmol_sum	5	0.938	0.938	0.119	0.179	0.958	0.111	0.143
norm_cont	6	0.964	0.964	0.070	0.137	0.988	0.052	0.077
fmol_diff	8	0.947	0.947	0.107	0.165	0.956	0.115	0.147
sqr_fmol	4	0.924	0.924	0.136	0.198	0.930	0.135	0.184
root_fmol	4	0.865	0.864	0.194	0.264	0.902	0.167	0.218
sqr_fmol_sum	5	0.954	0.954	0.093	0.154	0.976	0.090	0.108
**Deviation combinations**								
mol_dev	6	0.484	0.484	0.363	0.516	0.454	0.387	0.514
sqr_mol_dev	5	0.525	0.525	0.353	0.495	0.685	0.277	0.391
mol_dev_sqr	5	0.360	0.360	0.410	0.574	0.044	0.540	0.712
**Other combinations**								
cent	4	0.587	0.587	0.321	0.461	0.779	0.202	0.327
sqr_diff	6	0.590	0.590	0.325	0.460	0.776	0.219	0.330
abs_diff	6	0.600	0.600	0.311	0.454	0.768	0.223	0.336
